# Antibiotic Resistance: From the Bench to Patients, 2.0

**DOI:** 10.3390/antibiotics15060625

**Published:** 2026-06-22

**Authors:** Márió Gajdács, Fernando Albericio

**Affiliations:** 1 Department of Public Health, Albert Szent-Györgyi Faculty of Medicine, University of Szeged, 6720 Szeged, Hungary; 2 MTA-SZTE Lendület “Momentum” Anthropogenic Stress and Plant Resilience Research Group, Közép fasor 52, 6726 Szeged, Hungary; 3 School of Chemistry, University of KwaZulu-Natal, Durban 4001, South Africa; albericio@ukzn.ac.za; 4 Department of Inorganic and Organic Chemistry, University of Barcelona, 08028 Barcelona, Spain

## Introduction

The introduction of antibiotics into routine clinical practice in the 1950s marked a new era in medical care, allowing for the treatment of bacterial infections that were previously life-threatening or lethal [1]. Together with the widespread introduction of vaccines and infection prevention and control (IPC) measures, antibiotics have revolutionized healthcare and altered mortality patterns globally [2]. However, the widespread use of antimicrobials in subsequent decades has given rise to multidrug-resistant (MDR) bacteria, which often cause difficult-to-treat infections with few remaining treatment options. Antimicrobial resistance (AMR) has become a critical public health issue of the 21st century, further exacerbated by the SARS-CoV-2 pandemic. According to recent reports, AMR may surpass malignant diseases as the major cause of death in the next 50 years [3]. Given the concerns associated with AMR, international bodies, political actors (including those who have participated in a second high-level meeting on AMR in the United Nations General Assembly), pharmaceutical companies and other relevant stakeholders have notably committed to planning and carrying out actions needed to overcome this critical public health issue [4]. In addition, the disease burden of AMR disproportionally affects individuals in low- and middle-income countries (LMICs), further fueling inequalities globally [5]. As a result, several major developments were carried out globally, including commitments to national action plans (NAPs) for AMR, a heightened emphasis on antimicrobial stewardship (AMS) and AMR surveillance (such as the WHO “GLASS” project [6]), the rollout of the WHO “Access-Watch-Reserve” (AWaRe”) classification [7], and discussions on a more sustainable financing system for antimicrobial research and development.

The first edition of the Special Issue (SI) “Antibiotic Resistance: From the Bench to Patients” was published in 2020. It included nine excellent papers reporting on current results on the epidemiology of various bacterial pathogens—including trends in the infections caused by MDR bacteria—novel options for their diagnostics and susceptibility testing, and reports on the knowledge, attitude, and practices (KAP) of healthcare professionals (nurses, doctors, pharmacists, etc.) and patients regarding infectious diseases and AMR in the global context [8]. Given the success of the first edition, we were encouraged to open a second SI on the same topic, with a similar goal in mind. As a result, this SI comprises seven high-quality articles of diverse study types (including laboratory, epidemiological and clinical research) from 85 authors, spanning 14 different countries. Crucially, six out of seven papers are related to LMICs [Zambia (2), Vietnam, Pakistan, and Chile], aiming to enhance our understanding of the molecular, epidemiological, health-system-related and social dimensions of AMR and to provide an evidence base for future public health action.

There is an ever-increasing appreciation of the interconnected nature of AMR within the One Health context, whereby interventions in all respective domains (i.e., human health, animal health, agriculture, and the environment) are necessary to address this global issue. Kasanga et al. (Contribution 1) conducted a comprehensive genomic and phenotypic analysis of extended-spectrum β-lactamase (ESBL)-producing *Escherichia coli* (*E. coli*) across clinical and environmental reservoirs in Lusaka, Zambia. Their study framed the issue of AMR as a multi-sectorial and systemic issue driven by widespread and often inappropriate use of antimicrobials across all domains of the One Health framework, including human medicine, agriculture, and livestock production. Using whole-genome sequencing (WGS) on multidrug-resistant (MDR) *E. coli*, they demonstrated substantial genetic diversity alongside convergence in resistance determinants across human, animal, and environmental sources, including a high prevalence of ESBL-producing *E. coli* (31.8% among isolates), with predominance of phylogroup B2 and globally disseminated high-risk clones, particularly ST131 (O25:H4). Resistance against β-lactams was mainly associated with *bla*_CTX-M_-type enzymes—with broad resistomes, carrying resistance determinants to tetracyclines, as well as sulfonamides—while carbapenem susceptibility remained largely preserved, with occasional detection of the carbapenemase gene *bla*_NDM-5_. Their study demonstrated comparable resistance gene profiles and sequence types between clinical and environmental isolates, indicating ongoing cross-sectoral dissemination. In summary, their work provides robust genomic evidence for the interconnected spread of ESBL-producing *E. coli* in a low-resource setting and underscores the need for cross-cutting interventions, including integrated surveillance, AMS, and IPC strategies within a One Health framework to curb the expansion of high-risk clones and resistance genes.

Because of AMR, managing infections in immunocompromised hosts and individuals affected by malignant disorders has become a critical challenge, leaving these patient populations especially vulnerable to additional morbidities, with a higher risk of complications or death. Singer et al. (Contribution 2) aimed to provide single-center insights into the bacterial pathogen distribution and AMR patterns in pediatric leukemia patients with severe infections. The cohort (*n* = 30) predominantly comprised acute lymphoblastic leukemia cases and revealed a diverse pathogen spectrum with Gram-positive organisms (60%); methicillin-resistant *Staphylococcus aureus* (MRSA) was the most frequent pathogen (36.6%), alongside its methicillin-susceptible counterpart (MSSA), *E. coli*, and *Klebsiella* spp. These results highlight a high healthcare burden due to MDR infections. Resistance profiles demonstrated limited efficacy of commonly used β-lactams and macrolides, while susceptibility was preserved for “last-resort” antimicrobials, such as linezolid, teicoplanin, tigecycline, and carbapenems. Linezolid was highlighted for its relevance in this specific patient population due to its central role in managing resistant Gram-positive infections, owing to high susceptibility levels in MRSA (~91%) and its better pharmacokinetic/pharmacodynamics properties compared to other drugs against Gram-positive bacteria (e.g., glycopeptides and daptomycin). Due to the substantial heterogeneity observed in the resistance patterns across pathogens, the study emphasized the importance of continuous surveillance of pathogen profiles and resistance trends in pediatric oncology settings and the need for individualized, antibiogram-guided therapeutic strategies in the context of AMS.

Lower respiratory tract infections (LRTIs), including community-acquired pneumonia (CAP), remain a substantial cause of pediatric mortality in LMICs. Phuong et al. (Contribution 3) aimed to evaluate the clinical realities in managing CAP in children under five using a mixed-methods approach, within the context of a major Grade I hospital in Vietnam. Their quantitative analysis—involving the screening of 108 pediatric records—revealed that (i) many of the children had already received antibiotics before being admitted, complicating empiric treatment choice; (ii) most children (>90%) received their treatment intravenously, without much evidence of later step-down to oral administration of these drugs; (iii) adherence to national treatment guidelines was low during the prescription of initial antibiotic regimens, with patients often receiving dosages higher than those recommended; and (iv) despite both national and international (WHO AWaRe) guidelines recommending the use of narrower-spectrum agents, 73.3% of prescribed antibiotics belonged to the WHO “Watch” category, with third-generation cephalosporins being the most common choice. In addition, in-depth qualitative interviews were conducted with pediatricians, which identified various internal (e.g., physician habits) and external factors (e.g., supply chain issues), including outdated national CAP guidelines (which do not consider recent developments in AMR rates), inconsistent availability of certain medicines, and clinical complexity due to the high rate of prior antibiotic use in the community, leading to first-line drugs being insufficient to manage patients arriving at tertiary facilities. Based on their findings, the authors underscored the importance of updating national guidelines for clinicians (taking into consideration the WHO AWaRe guidelines) and the involvement of clinical pharmacists to aid in decision-making and training to improve antibiotic use in ambulatory care.

Diagnostic stewardship—a critical domain of AMS initiatives—represents an intersection between clinical practice and laboratory science, focusing on the accurate use and timely administration of diagnostic tests to guide therapeutic decisions, improve patient outcomes, and preserve the efficacy of existing drugs. Chizimu et al. (Contribution 4) conducted a retrospective analysis of diagnostic stewardship trends and rates of AMR in Zambia over a five-year period (2020–2024), during which data from seven sentinel surveillance sites and WHONET were utilized. Their analysis included data for over 180,000 bacteriological specimens associated with provincial hospitals, tertiary referral centers and university teaching hospitals. They observed a notable increase in diagnostic capacity and reporting across all sites over the five-year period (21,756 vs. 47,672 specimens cultured). The detected resistance rates were particularly concerning: non-susceptibility to commonly used drugs (e.g., azithromycin, clindamycin, trimethoprim/sulfamethoxazole, ciprofloxacin, and doxycycline) was between 50 and 80%, while rates of resistance against many last-resort agents (e.g., vancomycin-resistance in *Enterococcus* spp., linezolid resistance, and ESBL- and carbapenemase production in Enterobacterales) had reached critical levels. These findings provide much needed insights into the AMR landscape in Zambia, particularly from the human health perspective, signaling that empirical use of first-line antimicrobials may need to be re-evaluated due to high resistance rates. Furthermore, the authors described the urgent need for robust AMS and continuous diagnostic surveillance in hospital settings.

Ensuring that healthcare professionals have appropriate knowledge and attitudes towards infectious diseases and AMR is a key drivers in preventing the antimicrobial resistance crisis from worsening [9]. In contrast, insufficient KAP—together with a burdened healthcare system and specific societal norms associated with antimicrobials—may present a considerable risk for inappropriate prescribing and dispensing practices, in addition to self-medication, especially in LMICs [10,11]. Considering this, Shahid et al. (Contribution 5) explored the KAP of healthcare workers (*N* = 326) in Pakistan regarding AMR through a mixed-methods approach. Their study showed a notable disconnect between AMR-related knowledge, beliefs and practices: while the majority held favorable attitudes toward combating resistance, indicating a willingness to improve, over half (55.5%) of the respondents demonstrated insufficient knowledge regarding AMR and antimicrobial use, and nearly as many (48.5%) of them engaged in suboptimal prescribing practices; in addition, 49.4% were unaware of AMS programs existing in their setting. Based on these results, prior training in microbiology or infectious diseases was associated with better practices. Several main drivers—including pressures from patients to prescribe antimicrobials, an accepted culture of sharing medications among household members and friends, and weak AMR-related governance—were identified in the local context of Pakistan. Their study also introduced precision medicine (PM), a specific solution to combat AMR. The participants emphasized that PM could be particularly useful to treat resistant infections and to prevent the misuse of broad-spectrum antibiotics; however, they also acknowledged the lack of standardized clinical protocols, instrumentation, and funding and training of healthcare staff to implement PM, in addition to more pressing issues facing the healthcare system at present. Overall, the authors highlighted that investing in infrastructure (i.e., increasing access to diagnostic tools and improving microbiological capacity) would be a pre-requisite for the broad implementation of both diagnostic stewardship and PM.

NAPs are essential to benchmark the status of AMR in a given country, to outline priority actions to effectively combat AMR, and to measure progress associated with these initiatives [12]. Acuna et al. (Contribution 6) detailed the initiatives implemented in Chile to combat AMS, as well as the evolution of AMS programs in the country. The researchers initially defined AMS programs as multidisciplinary strategies, aiming to optimize therapy (including administration of the correct drug with the correct dose, route, and duration for the management of infections), improve outcomes, reduce the development of resistance, and contain therapeutic costs (including indirect reductions in healthcare expenditures by preventing inappropriate antimicrobial use). The paper describes initial developments in community-level AMS, including the introduction of regulations requiring medical prescriptions to purchase antibiotics (1999), and more recently, the establishment of guidelines to standardize outpatient infection management. Following WHO’s Global Action Plan on AMR, Chile has taken the “One Health” approach into account, implementing an Agricultural and Livestock Service (SAG) that restricts antibiotics critically important to human health, as well as an Electronic Prescribing System to be used by veterinarians. Considerable changes were also made in the hospital sector, such as the publication of technical standards to modernize hospital AMS programs (including stewardship metrics in mandatory institutional performance metrics for health services), the introduction of rapid diagnostic capacities (such as mass spectrometry and molecular biology), and the incorporation of the WHO AWaRe classification. Their summary also highlights several challenges, including shortages of specialized human resources and microbiological diagnostic capacity in the country (with substantial regional inequalities observed), in addition to the low level of consumption of “Access” group antibiotics (barely above 50%), falling short of established WHO goals.

Carbapenem-resistant *Acinetobacter baumannii* (CRAB) has been recognized as a Priority 1: Critical pathogen in the WHO’s Bacterial Priority Pathogen List (2024) due to its ability to target vulnerable hosts, persist in nosocomial environments, and develop resistance against most available antimicrobial drugs, and due to the high mortality rate associated with these infections [13]. Surveillance of resistance in priority pathogens is crucial to effectively track local epidemiological dynamics and to shape IPC and AMS efforts. The study of Chen and Er, conducted in a 450-bed regional teaching hospital in Central Taiwan, aimed to describe the temporal trends and ward-specific distributions of *A. baumannii* complex isolates (Contribution 7). Their retrospective, observational study highlighted a notable escalation in the rate of institutional CRAB burden, which increased from 44.0% to 63.9% between 2020 and 2021; furthermore, this rate remained between 58.3 and 63.2% through 2024, indicating that the spike in CRAB seen during the acute phases of the COVID-19 pandemic did not return to the pre-pandemic institutional baseline (2019: 46.0%). They also described sputum as the predominant specimen source (57.4%) and revealed that intensive care units (ICUs) contribute the single largest share (55.6%) of all CRAB isolates in this setting. Overall, they emphasized the need to invest in sustained surveillance and target ICU environments with strict contact precautions, enhanced environmental cleaning, and restricted carbapenem exposure—interventions which are relevant at the local, regional, and global levels—to address elevated CRAB isolation rates in the post-COVID-19 era.

Through seven rigorously selected studies spanning laboratory, epidemiological, and clinical research in LMICs, this Special Issue deepens the current understanding of the molecular, epidemiological, social, and health-system-related aspects of antimicrobial resistance while informing future public health strategies.

## References

[1] Aminov R.I. (2010). A Brief History of the Antibiotic Era: Lessons Learned and Challenges for the Future. Front. Microbiol..

[2] Asadulghani M., Griffith N.K., Arndt W. (2026). Containment of antimicrobial resistance for strengthening global public health security: Biorisk management perspectives. Biosaf. Health.

[3] (2024). GBD 2021 Antimicrobial Resistance Collaborators. Global burden of bacterial antimicrobial resistance 1990–2021: A systematic analysis with forecasts to 2050. Lancet.

[4] Hsu L.Y., Legido-Quigley H., Chua A.Q. (2025). High-level political commitment to action against AMR: What is next?. BMJ Public Health.

[5] Ehsan H. (2025). Antibiotic Resistance in Developing Countries: Emerging Threats and Policy Responses. Public Health Chall..

[6] WHO Global Antimicrobial Resistance and Use Surveillance System (GLASS). https://www.who.int/initiatives/glass.

[7] The WHO AWaRe (Access, Watch, Reserve) Antibiotic Book. https://www.who.int/publications/i/item/9789240062382.

[8] Gajdács M., Albericio F. (2019). Antibiotic Resistance: From the Bench to Patients. Antibiotics.

[9] Lagadinou M., Tsami E., Deligakis A., Paraskevas T., Michailides C., Velissaris D., Gkentzi D., Marangos M. (2023). Knowledge and Attitudes of Healthcare Workers towards Antibiotic Use and Antimicrobial Resistance in Two Major Tertiary Hospitals in Western Greece. Antibiotics.

[10] Jamromi A.S., Namavari N., Jokar M., Sharifi N., Soleimanpour S., Naserzadeh N., Rahmanian V. (2025). Global knowledge, attitudes, and practices towards antimicrobial resistance among healthcare workers: A systematic review and meta-analysis. Antimicrob. Resist. Infect. Control.

[11] Aslam A., Gajdács M., Zin C.S., Rahman N.S.A., Ahmed S.I., Zafar M.Z., Jamshed S. (2020). Evidence of the Practice of Self-Medication with Antibiotics among the Lay Public in Low- and Middle-Income Countries: A Scoping Review. Antibiotics.

[12] Charani E., Mendelson M., Pallett S.J.C., Ahmad R., Pallett S.J.C., Ahmad R., Mpundu M., Mbamalu O., Bonaconsa C., Nampoothiri V. (2023). An analysis of existing national action plans for antimicrobial resistance—Gaps and opportunities in strategies optimising antibiotic use in human populations. Lancet Glob. Health.

[13] WHO Bacterial Priority Pathogen List (BPPL). https://www.who.int/publications/i/item/9789240093461.

